# The Informative Process Model as a New Intervention for Attitude Change in Intractable Conflicts: Theory and Empirical Evidence

**DOI:** 10.3389/fpsyg.2022.946410

**Published:** 2022-07-26

**Authors:** Nimrod Rosler, Keren Sharvit, Boaz Hameiri, Ori Wiener-Blotner, Orly Idan, Daniel Bar-Tal

**Affiliations:** ^1^Program in Conflict Resolution and Mediation, Tel Aviv University, Tel Aviv, Israel; ^2^Department of Psychology, University of Haifa, Haifa, Israel; ^3^School of Psychology, Reichman University, Herzliya, Israel

**Keywords:** intractable conflict, attitude change, narratives, Israeli-Palestinian conflict, peace

## Abstract

Peacemaking is especially challenging in situations of intractable conflict. Collective narratives in this context contribute to coping with challenges societies face, but also fuel conflict continuation. We introduce the Informative Process Model (IPM), proposing that informing individuals about the socio-psychological processes through which conflict-supporting narratives develop, and suggesting that they can change via comparison to similar conflicts resolved peacefully, can facilitate unfreezing and change in attitudes. Study 1 established associations between awareness of conflict costs and conflict-supporting narratives, belief in the possibility of resolving the conflict peacefully and support for pursuing peace among Israeli-Jews and Palestinians. Studies 2 and 3 found that exposure to IPM-based original videos (vs. control) led Israeli-Jews to deliberation of the information presented, predicting acceptance of the IPM-based message, which, in turn, predicted support for negotiations. Study 3 also found similar effects across IPM-based messages focusing on different conflict-supporting themes. We discuss the implications to attitude change in intractable conflicts.

## Introduction

Promoting peaceful resolution of intergroup conflicts is especially challenging in situations of intractable conflict, in which attitudes are deeply entrenched and frozen (e.g., Paluck, 2012; Tropp, 2015; Bar-Tal and Hameiri, 2020). Conflicts of this type are violent and protracted; demand extensive investment; play a central role in the lives of individuals in the involved societies; are fueled by conflict supporting narratives; and are perceived by those affected as total, irresolvable, and of a zero-sum nature (Coleman, 2003; Kriesberg, 1993; Bar-Tal, 2013).

Given their detrimental consequences, resolving intractable conflicts peacefully is one of civilization’s most important challenges because they still rage around the world, including in Kashmir, in the Middle East between Palestinians and Israelis, and in Turkey. Therefore, members of societies involved in intractable conflicts must find ways to change the conflict supporting narratives and to promote support for conciliatory attitudes, which may translate into policies that could later lead to a peacemaking process. While we recognize that different approaches attempt to meet this challenge (see e.g., Bar-Tal and Hameiri, 2020), in the current research, we delineate one new approach for intervention to promote change toward more conciliatory attitudes, based on the *informative process model* (IPM). This intervention is based on the comprehensive, systematic and holistic theory of development of intractable conflict that focuses on conflict-supporting narratives, the functions they fulfill, and their role in fueling the conflict and becoming barriers to peaceful conflict resolution (Bar-Tal, 2013 see also Auerbach, 2010; Hammack, 2011). The IPM-based intervention is designed to specifically address these conflict-supporting narratives.

### Conflict-Supporting Narratives: Their Freezing and Consequences

According to different accounts on the psychological foundations and dynamics of intractable conflicts (Kriesberg, 2005; Coleman, 2006; Kelman, 2007; Bar-Tal, 2013), all societies involved in such conflicts must function for extended periods under conditions of intense threats, deprivation of needs, stress, hardship, and resource austerity. To adapt to the difficult conditions and cope with the challenges that the conflicts pose, members of these societies form collective narratives that support their continuation. These narratives include societal beliefs referring, among other themes, to the justness of the ingroup’s goals, the importance of security, delegitimization of the rivals, ingroup glorification and victimization, and yearning for peace (Eidelson and Eidelson, 2003; Hadjipavlou, 2007; Papadakis, 2008; Hammack, 2009; Garagozov, 2012; Bar-Tal, 2013).

The developed narratives serve important functions for individual and collective needs, such as justifying the goals of the conflict, finding meaning in conflict events, legitimizing aggressive actions by the ingroup, differentiating between the ingroup and the rivals, and mobilizing society members to engage in the conflict (Lederer et al., 1980; Burton, 1990). Therefore, such societies devote great efforts to imparting these narratives to their members and maintaining them through time (Hammack, 2011; Bar-Tal, 2013; Oren, 2019). With time, due to their functionality, the narratives often become hegemonic, widely shared, deeply entrenched and frozen (Maoz and McCauley, 2008; Bar-Tal and Halperin, 2011; Vollhardt and Bilali, 2015; Rosler et al., 2018). The latter denotes a preference for maintaining the hegemonic narratives and resisting their change, leading to the rejection of alternative, peace-supporting information (Kruglanski and Webster, 1996; Peterson and Flanders, 2002; Kruglanski, 2004). The continuation of the conflict goes together with immense costs to the involved societies in terms of loss of life, destruction, suffering, mental stress, and tangible and non-tangible investment (Brubaker and Laitin, 1998; Lake and Rothchild, 1998; Kelman, 2007).

Importantly, individuals living in these contexts of conflict are usually not aware that these processes are taking place, namely, that narratives develop to serve specific needs and consequently freeze, and that similar dynamics have been and are occurring in other past and present intractable conflicts. Instead, most individuals cling to the conflict-supporting narratives due to their functionality, while accepting the continuation of the conflict and its immense costs as inevitable and even necessary. Considering the possibility of change to the ongoing conflict creates ontological insecurity, anxiety and resistance due to concerns about uncertainty and risk taking (Mitzen, 2006; Marcus, 2014; Rumelili, 2014; Elman et al., 2019; Kossowska et al., 2020). Therefore, policies aimed at peaceful conflict resolution that require changing conflict-supporting narratives (Clarke-Habibi, 2005; Papadakis et al., 2006; Paluck, 2009; Kriesberg and Dayton, 2016; Garagozov and Gadirova, 2019) are often rejected as non-relevant, impossible to implement and even treacherous.

Through the years, social scientists have devoted much effort to developing interventions to meet the challenge of changing conflict supporting narratives, assuming that such a process could advance peacebuilding among rival societies. Different interventions have been developed, based on different principles and approaches (e.g., Al Ramiah and Hewstone, 2013; Ditlmann et al., 2017; Bar-Tal and Hameiri, 2020). The vast majority of peace-promoting interventions attempt to induce inconsistency by providing information that negates the existing conflict-supporting narratives directly or indirectly (e.g., Gayer et al., 2009; Girod et al., 2016). Another approach, is based on teaching new skills to help individuals overcome emotional or cognitive limitations that prevent exposure to alternative information (Halperin et al., 2014; Alkoby et al., 2017); An additional approach—*paradoxical thinking*—uses messages that are *consistent* with the targeted audience’s held beliefs, but are provided in an amplified, exaggerated, or even absurd manner with the aim of creating deliberation and change (Hameiri et al., 2019). Thus, in comparison with messages that attempt to induce inconsistency, these paradoxical thinking messages do not trigger strong disagreement, resistance, or psychological defenses; rather, they raise a threat to the identity of the message recipient, and in turn induce re-evaluation of held beliefs and attitudes (Bar-Tal et al., 2021).

While these various interventions have yielded promising results, they have mostly been examined in small groups, in the lab or in the field, and are logistically difficult to scale up for the masses. This is due both to the practical constraints of bringing people from enemy groups to meet and to the lack of motivation of members in societies immersed in intractable conflicts to participate and be exposed to these interventions, as a result of intense intergroup mistrust and powerful socialization into conflict-supporting beliefs. This was found to be particularly important for contact interventions (Lemmer and Wagner, 2015; Tropp, 2015; Paolini et al., 2018; Salma, 2020), but is relevant to other interventions as well (Bar-Tal and Hameiri, 2020).

Moreover, we argue that, in general, these approaches do not take into account the full scale of psychological dynamics that unfold in contexts of intractable conflict, with the entrenched beliefs and narratives, and the unfulfilled needs that underlie individuals’ resistance to changing their dysfunctional and costly behaviors (e.g., Bar-Tal and Halperin, 2011; Hornsey and Fielding, 2017). In the present research, we present a novel intervention, the informative process model (IPM), that focuses on these specific underlying beliefs, narratives, and needs, in order to lead to change, and based on the theory of intractable conflict (Bar-Tal, 2013).

### The Informative Process Model

**FIGURE 1:**
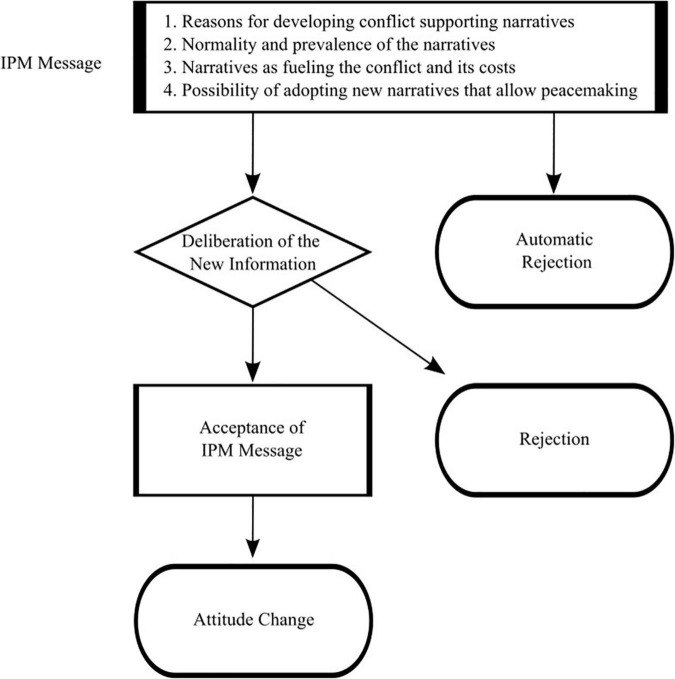
The Informative Process Model (IPM) and its hypothesized effects.

The IPM suggests that change in beliefs can occur by informing individuals about the socio-psychological processes through which they form and maintain their beliefs in the context of intractable conflict. The proposed intervention is based on messages that explain the socio-psychological process of forming conflict-supporting narratives, elaborating the four following elements (see Figure 1): 1. the reason for the evolvement of conflict-supporting narratives, namely, to fulfill the primary human needs of society members; 2. the prevalence and normality of such narratives in every society involved in intractable conflict to fulfill the same needs; 3. the immense costs that societies pay for the continuation of the intractable conflict and the role of the conflict-supporting narratives in fueling the conflict and serving as barriers to conflict resolution, thus contributing to the costs; and 4. the possibility of changing the conflict-supporting narratives with peace-supporting narratives that can also satisfy the same primary human needs.

Informative Process Model-based messages are conveyed by informing recipients of accumulated knowledge of historical and present-day conflicts, in which functional but costly conflict-supporting narratives developed, but eventually changed and enabled peaceful conflict resolution that reduced the costs. The comparison to other conflicts that have been resolved helps demonstrate that the process through which narratives revolving around intractable conflicts are constructed is prevalent, normal and functional, thus expressing understanding and acceptance of the recipients’ views. At the same time, learning that other conflicts with similar characteristics have been resolved peacefully helps convey the message that change and progress toward conflict resolution are possible. The combined communication of acceptance and change helps deliver the message in a non-threatening way that may facilitate unfreezing of individuals’ narratives regarding the intractable conflict and the means to resolve it.

The IPM draws from the literature on attitude change as well as the literature on clinical psychotherapy. The first and fourth elements of the IPM that refer to the reasons for the development of the narratives and the possibility of changing the latter, respectively, are partially based on the relationship between needs and attitudes. During the 1950’s functional theories of attitudes appeared, suggesting that attitudes serve various psychological needs and thus have a motivational basis (Smith et al., 1956; Katz, 1960; Eagly and Chaiken, 1993). Although each of the theories focused on four general needs as utilitarian needs, knowledge needs, ego-defensive needs, and value expressive needs, we relied on needs proposed by Maslow in his motivation theory (Maslow, 1970). Of special importance are those needs that are deprived from society members in this context: safety needs that are satisfied when they live in a secure environment; a need for mastery that leads them to strive for predictability and a sense of self-control; and a need for positive self-evaluation, respect, and esteem as individuals and members of a society (Maslow, 1970).

Furthermore, extensive research on attitude change has shown that when confronted with counter-attitudinal messages, individuals may experience threat and become motivated to defend their existing attitude (Cacioppo and Petty, 1979; Chen and Chaiken, 1997). In addition, individuals may be motivated to bolster their existing attitudes (Clark and Wegener, 2013). Accordingly, individuals may engage in various motivated reasoning processes in order to maintain their existing attitudes and beliefs and avoid changing them (Kunda, 1990; Taber and Lodge, 2006), such as selective information processing (Clark et al., 2008) or generating counter-arguments (Cacioppo and Petty, 1979; Eagly et al., 2000). Consequently, counter-attitudinal persuasive attempts may not always be effective and at times may even result in strengthening existing attitudes and beliefs (Miller, 1993; Kuhn and Lao, 1996; Hart and Nisbet, 2012). This led Hornsey and Fielding (2017) to propose that messages that address the motivations underlying individuals’ beliefs without directly contradicting these beliefs could enhance persuasiveness. Indeed, studies have found that framing scientific information in a way that resonated with recipients preexisting values and beliefs increased its persuasiveness (Luong et al., 2019; Fielding et al., 2020). In addition, two-sided messages, which address the opposing viewpoint in addition to the advocated position, have been found to increase persuasion under certain circumstances (Kao, 2011), and importantly, to increase openness toward alternative viewpoints (Xu and Petty, 2021). Finally, messages that originate from in-group members or from similar others were found to create willingness to listen, engage and change attitudes (Kelman, 1958; Turner and Oakes, 1986). These findings led us to believe that the combinations of elements that accept conflict-supporting narratives by presenting them as normal and functional, and elements that challenge these narratives by pointing out their costs and suggesting that change is possible, originating from a source that may induce context-based identification could enhance the persuasiveness of IPM-based messages.

Interestingly, some of the scholars who discussed the importance of addressing existing attitudes and beliefs to enhance persuasiveness (Hornsey and Fielding, 2017; Xu and Petty, 2021) noted certain similarities between these approaches and techniques used in clinical psychotherapy, which seek to promote change of dysfunctional and costly patterns. We noted these similarities as well and relied on them in designing our IPM-based intervention. Specifically, modern therapeutic techniques tend to have an approach of openness and acceptance toward the views and perceptions of people, even if they seem to be “irrational” (Hayes et al., 2004b). In some cases, people’s perceptions comprise a part of themselves, and to reject these perceptions is to reject a part of them. Therefore, within the context of clinical psychotherapy, it is critical that the client feels validated for his/her perceptions, and a sense of complete acceptance by the therapist. Only then can a therapist challenge those same existing beliefs to motivate change (Omer and Elitzur, 2001). It is also important to find a balance between acceptance and change. With too great a focus on acceptance, the client may not be motivated to grow, and with too great a focus on change, the client may feel misunderstood or criticized, which can hinder change rather than promote it (Brodsky and Stanley, 2013). These therapeutic techniques show that individuals are more likely to be open to hearing about the potential benefits of alternative points of view and their acceptance when they think that they are understood, and their feelings are viewed as legitimate. Their readiness to change increases when they realize that they are paying high costs for their beliefs, while the costs for alternative beliefs are lower. Dialectical Behavioral Therapy (DBT), for example, is an extensive manualized treatment in which the acceptance-change dialectic is a core principle (Linehan, 2015). Dialectical thinking was found to be associated with more cognitive flexibility, and less anxiety (Cheng, 2009). This acceptance-change dialectic process occurs when a psychologist accepts patients with empathy and understanding, but also encourages them to make changes in their dysfunctional system of beliefs (Arlo, 2017). DBT has outperformed other forms of treatment in randomly controlled trials across different populations in measures such as higher rates of treatment completion, lower rates of various forms of self-harm, and less reported depression, anger, and hopelessness (Hayes et al., 2004a).

While we acknowledge that the context of clinical psychotherapy is very different from the context of intervening to change conflict-supporting narratives, we argue that lessons can be applied to the context of intractable conflict. We base our argument on the similarity of some principles of psychotherapeutic interventions to those noted by attitude change scholars (Hornsey and Fielding, 2017; Xu and Petty, 2021), and to previous prejudice reduction and conflict resolution interventions such as paradoxical thinking (Bar-Tal et al., 2021) and emotion regulation (Halperin et al., 2013). In particular, to implement the acceptance-change dialectical approach in the context of intergroup conflicts, it is necessary to convey acceptance to society members by expressing an understanding of how their narratives were formed and indicating that they are normative. In addition, it is important to encourage change by demonstrating that these narratives can lead to costly behavior. These principles, adapted to the collective level, are reflected in the four elements of the IPM-based intervention that we developed and are conveyed through the comparison to similar conflicts that have been resolved.

We thus hypothesize that messages that follow the IPM principles will lead to a process of unfreezing, which will ultimately result in increased support for policies aimed at resolving the conflict peacefully. Specifically, and as can be seen in Figure 1, unfreezing requires slow deliberative-cognitive processing rather than a fast, automatic, intuitive processing that usually ends with rejection of the message (Sloman, 2002; Evans, 2008; Kahneman, 2011). The deliberation process begins with consideration of the new information provided by the messages. This information acknowledges the normality and the need for conflict supporting narratives in societies involved in intractable conflicts, while suggesting that change is possible. Such deliberation should lead to acceptance of the gist of the IPM-based message, which will enable individuals to realize the cost and dysfunctionality of their current held beliefs. This process, in turn, can lead to increased support for policies that may advance peaceful conflict resolution.

### The Present Research

In the present research, we aimed to test the proposed IPM and validate the effectiveness of messages based on it. We believe that this particular process of narrative change through the IPM, which has not been conceptualized or researched until now, provides a new glimpse into the dynamics of change in beliefs within intractable conflicts that may direct attention to a new and effective intervention. The research examines our predictions in the context of the Israeli-Palestinian conflict, which serves as a typical intractable conflict. Its intractability is well reflected in the recent violent events related to the conflict that took the lives of 367 Palestinians and 17 Israelis in the last year alone.^1^ In addition to the grave loss of lives within the conflict, the two involved societies pay tremendous costs related to mental health, economy and democratic principles to name only a few areas (Bar-Tal and Raviv, 2021; Rosler et al., 2021). We decided to focus on two themes of the conflict-supporting narratives: justness of the goals and delegitimization of the rival. These two themes are central in the conflict supporting narratives. The first establishes the existential and sacred goals within the conflict that must be achieved, and rejects compromises (Tetlock, 2003; Ginges and Atran, 2011). The second excludes the rivals from the sphere of human groups and provides psychological permission to harm them and to reject them as partners for negotiations (Bar-Tal and Hammack, 2012; Haslam and Loughnan, 2014; Kteily et al., 2015). These two themes stand as major barriers to peacemaking processes and in reality, make them impossible.

We conducted a series of three studies. The first correlational study was administered to samples of Israeli Jews and Palestinians from the West Bank and the Gaza Strip. The aim of the study was to test our underlying assumption that understanding the costs of conflict and the role of conflict-supporting narratives in conflict continuation would be related to increased belief in the possibility of resolving the Israeli-Palestinian conflict, similar to other conflicts that had been resolved. These beliefs were expected to be related to support for pursuing future peaceful relations between Israelis and Palestinians. The next two studies were experiments conducted among Israeli Jews, in which IPM-based interventions were administered to test their effectiveness. The IPM-based messages were delivered via original short videos, carefully constructed in collaboration with an advertising agency. Two videos dealt with the justness of the goals and two videos dealt with delegitimization. In Study 2, we tested the general effectiveness of the intervention. In Study 3, we sought to replicate and validate the results of Study 2, and examine whether videos addressing one specific theme were more or less effective than videos combining the two themes, and whether one theme was more effective than the other. The objective of these studies was to establish the validity of the IPM intervention, as the first phase of a long and comprehensive program of research.

## Study 1

The aim of Study 1 was to provide preliminary support to some of the assumptions informing our IPM-based intervention. Specifically, we sought to investigate the extent to which there is a general awareness and understanding among Israelis and Palestinians of the costs of intractable conflicts and the associated socio-psychological processes, namely the development of conflict-supporting narratives. To the extent that some individuals display such awareness even in the absence of intervention, we were interested in whether it would be associated with believing in the possibility of resolving the Israeli-Palestinian conflict peacefully, based on its similarity to other conflicts that had been resolved, and by implication, to support for pursuing peaceful relations between Israelis and Palestinians. Revealing these correlations could provide preliminary support to the development of IPM-based interventions aiming to increase awareness and understanding of the development of conflict-supporting narratives and facilitate their change.

### Method

#### Sampling and Procedure

Measures of the variables of interest were added to a joint poll conducted simultaneously among representative samples of Israeli Jews and Palestinians between August 12 and September 3, 2020. Israeli Jewish respondents were recruited from an online pool managed by the Israeli polling company Midgam. Participants’ demographics, including gender, age, and religiosity, were collected in advance by the polling company. Cross-tabulating this information with demographic reports published by Israel’s Central Bureau of Statistics (2020) created a sample that represented the Israeli population on selected demographic criteria as age, gender, level of religiosity, and political orientation.

The Israeli sample included 704 Jewish respondents in total, of which 503 resided within the 1967 Green Line borders of Israel and 200 were settlers residing in the West Bank. Over-sampling of the settler population was needed for other purposes of the joint poll, which were unrelated to the present study. To accurately represent the population of Jewish citizens of Israel (Central Bureau of Statistics, 2020), we applied a weight of 0.17 to the settler sample and a weight of 1.33 to the rest of the sample in all subsequent analyses. The sample included 361 women (51%) and 342 men, aged 18–82 (*M*_*age*_ = 43.66, *SD*_*age*_ = 16.60). After weighting, the distribution of religious and political orientations resembled the Israeli-Jewish population. Regarding religiosity, 42% described themselves as secular, 32% as traditional, 14% as religious and 12% as Ultra-Orthodox. In terms of political orientation, 60% identified as rightists, 24% as centrists, 13% as leftists, and 3% did not report political orientation.

Palestinian participants were interviewed face-to-face by the Palestinian Center for Policy and Survey Research. To ensure a representative sample, the West Bank, East Jerusalem and Gaza Strip were divided into several strata, each representing the towns, cities, villages, and refugee camps in the 16 governorates (muhafazat), as well as clusters containing 80 to 200 families each. A sample of 120 clusters was randomly selected using probability proportionate to size, taking into consideration geographic region and strata. Within each cluster, 10 homes were sampled randomly, and within each home, a person who was 18 years or older was selected using Kish tables. The final sample included 1200 Palestinian respondents in total, of which 790 were residents of the West Bank (including East Jerusalem) and 410 were residents of the Gaza Strip. The sample included 625 women (52%) and 575 men, aged 18–90 (*M*_*age*_ = 41.61, *SD*_*age*_ = 15.10). Muslims constituted 99% of the sample and the remaining 1% were Christians. In terms of level of religiosity, 49% percent identified as very religious, 48% as somewhat religious, and 3% as not religious.

#### Measures

Unless indicated otherwise, all measures used a scale ranging from 1 = *not at all* to 6 = *to a great extent*.

*Awareness of conflict costs and conflict-supporting narratives* was measured using four items developed for the purpose of the current research, assessing the extent to which participants agreed with statements referring to the contents of conflict-supporting narratives, the reasons for their development and their implications (see Table 1). The items referred to processes taking place in conflicts in general, without reference to the specific Israeli-Palestinian context.

**TABLE 1:** Means, SDs and factor loadings of items referring to awareness of conflict costs and conflict-supporting narratives and belief in the possibility of resolving the Israeli-Palestinian conflict peacefully among Israelis and Palestinians (Study 1).

	Israelis				Palestinians			
Item	*M*	*SD*	Factor loadings		*M*	*SD*	Factor loadings	
			Awareness of conflict costs and conflict-supporting narratives	Belief in the possibility of resolving the conflict peacefully			Awareness of conflict costs and conflict-supporting narratives	Belief in the possibility of resolving the conflict peacefully
Perceptions that people develop in conflicts regarding their goals and their enemy escalate the violence	4.19	1.09	**0.752**	0.025	3.86	1.15	**0.803**	0.015
Following people’s experiences in all bloody and lasting conflicts, each party naturally perceives its goals as absolutely just and the enemy as inhumane.	3.98	1.13	**0.736**	−0.127	3.75	1.23	**0.709**	−0.088
People in all bloody and lasting conflicts pay tremendous costs for their continuation	4.75	1.22	**0.599**	0.197	4.09	1.12	**0.685**	0.28
Similar to other bloody and lasting conflicts that have been resolved, our conflict can also be resolved peacefully	3.50	1.34	−0.100	**0.903**	2.86	1.32	0.007	**0.740**
Similar to other bloody and lasting conflicts, changing our perception that Palestinians/Israelis are inhumane and that our goals are absolutely just can promote the peaceful resolution of our conflict	3.42	1.33	0.011	**0.720**	2.76	1.29	−0.075	**0.698**
Conflicts can be resolved if people change their perceptions about their goals and their enemy	4.00	1.14	0.336	0.506	3.37	1.25	0.384	0.421

*Items with loadings greater than 0.50 are in bold.*

*Belief in the possibility of resolving the Israeli-Palestinian conflict peacefully* was assessed using two items developed for the present research. The items referred to the possibility of resolving the Israeli-Palestinian conflict peacefully given its similarity to other prolonged and bloody conflicts that had been resolved (see Table 1).

*Support for pursuing peace* was assessed using one item asking the respondents about possible ways to handle Israeli-Palestinian relations from the present onward (i.e., “From the following possibilities, which do you prefer to do now regarding Israeli-Palestinian relations?”). Participants were given four options to select from, presented in random order: one option referred to maintaining the *status quo* (i.e., “maintain the situation as it is now”), one option to pursuing peace (i.e., “reach a peace agreement with the Palestinians\Israel”), and two options to conflict-escalating policies (for Israelis, annexation of the West Bank or parts of it, or a decisive war to destroy Palestinian military capabilities; for Palestinians, an unarmed or armed struggle against the Israeli occupation). There was also an option to indicate “other” if a different path forward was preferred. The scale was dichotomized to the one option supporting the pursuit of peace vs. all other options.

### Results and Discussion

Table 1 presents descriptive statistics of Israelis’ and Palestinians’ responses to the statements reflecting awareness of conflict costs and conflict-supporting narratives and the belief in the possibility of resolving the Israeli-Palestinian conflict peacefully. Israelis’ ratings of all items were higher than those of Palestinians (all *t*s > 4.00, all *p*s < 0.001). Among both groups, awareness of conflict costs and conflict-supporting narratives was generally greater than belief in the possibility of resolving the Israeli-Palestinian conflict peacefully, given its similarity to other conflicts that had been resolved. Regarding support for pursuing peace, less than half of the respondents (41% of Israeli Jews and 37% of Palestinians) indicated that reaching a peace agreement with the other party was their preferred option for handling Israeli-Palestinian relations.^2^

To affirm the distinction between our measures of awareness of conflict costs and conflict-supporting narratives and belief in the possibility of resolving the Israeli-Palestinian conflict peacefully, we conducted exploratory factor analyses in the Israeli and Palestinian samples with six items using maximum likelihood extraction and oblimin rotation. The analysis yielded similar two-factor solutions in both samples. Among Israelis, the first factor accounted for 38.39% of the variance and included three items with loadings greater than 0.50 referring to belief in the possibility of resolving the Israeli-Palestinian conflict peacefully. However, one of the items (i.e., “Conflicts can be resolved if people change their perceptions about their goals and their enemy”), originally intended to be part of the measure of awareness of conflict costs and narratives, had a cross-loading of 0.34 on the second factor. The second factor accounted for 17.08% of the variance and included three items with loadings greater than 0.50 referring to awareness of conflict costs and conflict-supporting narratives. Among Palestinians, the first factor accounted for 34.89% of the variance, and included three items with loadings greater than 0.60 referring to awareness of conflict costs and conflict-supporting narratives. The second factor accounted for 15.73% of the variance and included three items with loadings greater than 0.40 referring to belief in the possibility of resolving the Israeli-Palestinian conflict peacefully. However, one item (the same one as in the Israeli sample) had a cross-loading of 0.38 on the first factor. Table 1 shows all the factor loadings.

Because one item had cross-loadings on two factors in the two samples, we decided to exclude it from further analyses. The remaining items assessing awareness of conflict costs and conflict-supporting narratives had good internal consistency (α = 0.74 among Israelis, α = 0.78 among Palestinians), and the items referring to belief in the possibility of resolving the Israeli-Palestinian conflict peacefully were also strongly correlated (*r* = 0.63 among Israelis, *r* = 0.50 among Palestinians). Accordingly, we created indices for the two factors by averaging the respective items, and examined the correlation among all the variables in Study 1 (see Table 2). Among both Israelis and Palestinians, support for pursuing peace was positively correlated with awareness of conflict costs and conflict-supporting narratives and with belief in the possibility of resolving the Israeli-Palestinian conflict peacefully. In addition, awareness of conflict costs and conflict-supporting narratives was positively correlated with belief in the possibility of resolving the Israeli-Palestinian conflict peacefully.

**TABLE 2:** Bivariate correlations among Study 1 variables.

	Israelis (*N* = 704)			Palestinians (*N* = 1200)		
	1	2	3	1	2	3
1. Support for pursuing peace	1	–	–	1	–	–
2. Awareness of conflict costs and conflict-supporting narratives	0.12***	1	–	0.19***	1	–
3. Belief in the possibility of resolving the Israeli-Palestinian conflict peacefully	0.46***	0.27***	1	0.19***	0.49***	1

***p < 0.001.

Our findings support the hypothesized relationships between awareness of conflict costs and conflict-supporting narratives, belief in the possibility of resolving the Israeli-Palestinian conflict peacefully given its similarity to other conflicts that had been resolved, and support for pursuing future peaceful relations between Israelis and Palestinians. One limitation of the study is that the statements used in our measures were complex and involved multiple components. Participants may have responded only to certain parts of the statements and not to all the components. However, the factor analysis, which yielded similar results for Israelis and Palestinians, confirmed that our measures were tapping two distinct constructs that largely overlapped with the concepts we intended to assess. Having obtained preliminary support for some of the assumptions underlying the IPM, we proceeded to test the effectiveness of an intervention based on the IPM.

## Study 2

Study 2 was the first experimental examination of the IPM as a new intervention for attitude change toward supporting peacemaking in the Israeli-Palestinian intractable conflict. We hypothesized that the IPM-based intervention would lead to unfreezing by first engaging participants deliberatively with the new information provided in the messages. Deliberation would increase acceptance of the main arguments of the IPM-based message, namely, the normality and necessity of conflict-supporting narratives, specifically involving delegitimization of the rival and justifying the ingroup’s goals in the context of intractable conflicts, as well as the dysfunctionality of these beliefs for resolving these conflicts. Through this process of unfreezing, the intervention would have a downstream effect on participants’ support for policies aimed at peaceful resolution of the Israeli-Palestinian conflict.

### Method

#### Participants

Five hundred and two Israeli Jews completed the survey through an online surveying company, Midgam (*M*_*age*_ = 39.09, *SD*_*age*_ = 13.13; 50.4% women). In terms of political orientation, the sample resembled the Israeli-Jewish population, as 64.7% were rightists, 22.9% were centrists, and the remaining 12.4% were leftists. In exchange for participation, participants received 4 ILS (equivalent to 1.1$). Sensitivity power analysis indicated that our sample size allowed us to detect an effect size of Cohen’s *d* = 0.25, with 80% power when comparing two independent groups.

#### Procedure and Materials

Participants were asked to take part in a study in which they would watch a short video and respond to some questions. They were then randomly assigned to one of two conditions. Participants in the *IPM* condition (*n* = 237) were asked to watch a short 3-min video containing four 40-s clips in Hebrew entitled “How Conflicts End”. The videos followed the IPM principles, in that they presented elements of conflict-supporting narratives and their functionality, focusing on outgroup delegitimization and the justness of the ingroup’s goals (Bar-Tal et al., 2012; Bar-Tal, 2013). This was done using examples from conflicts taking place around the world without mentioning the Israeli-Palestinian conflict directly, while showing that the other presented conflicts had ended peacefully. Each video focused on a different historical conflict, namely, the Northern Ireland conflict, French-Algerian war, Spanish-Basque conflict, and the Guatemalan civil war. None of the videos explicitly mentioned the Israeli-Palestinian conflict, but they all led the audience to think about it by presenting explicit and implicit cues and statements expressing familiar narratives about the Israeli-Palestinian conflict. The videos showed persons who were covered, with only their eyes exposed, describing the other conflicts and thus, misleading the viewers to think that they were Israelis, until their true identity was revealed. Only toward the end of the video were the viewer’s aware that the person was a foreigner referring to a different conflict that had ended peacefully (see videos at https://youtu.be/PDeshDBVT9g). In the *Control* condition, participants (*n* = 265) watched a 3-min video, which contained four generic television commercials unrelated to intergroup relations.

After watching the video, participants were asked to answer four multiple choice attention verification questions—one for each video (in the experimental condition we asked, e.g., “How did the conflict in the second video end?”, “To which conflict did the third video refer?”; in the control condition we asked, e.g., “Which coffee did the second commercial try to sell?”, “What type of drink is depicted in the third commercial?”). Participants who answered these questions correctly continued to complete the dependent variables questionnaire, which included the measures detailed below in a fixed order, as well as some additional exploratory measures (for complete materials and data for Studies 2 and 3, as well as the additional measures see https://osf.io/qr6jn/?view_only=f0dedb3658a24e9c86f351e9ec03a4fc).

#### Measures

*Deliberation of new information* was measured using five items (α = 0.88) assessing the extent to which the videos made participants engage deliberately with the information conveyed in general (e.g., “Please rate the extent to which the videos made you think deeply about the conveyed messages”), and with regard to the Israeli-Palestinian conflict in particular (Hameiri et al., 2018). Four items were measured on a 1–6 scale and one item on a 0–100 scale. Therefore, the items were standardized before computing a composite score.

*Acceptance of IPM-based message* regarding conflict-supporting narratives and their implications was measured using three items (α = 0.71) similar to those used in Study 1, but adjusted to the specific narrative themes of justness of the goals and delegitimization of the rival that we used in the videos (i.e., ‘‘Dehumanizing views of the enemy develop due to conflict-related events,’’ ‘‘Views that each side in the conflict develops about the ingroup and the rival intensify the conflict,’’ and ‘‘Conflicts can end if views about the conflict and the rival are changed’’).^3^

*Support for negotiation* was measured using three items (α = 0.86) assessing participants’ support (from 1 = *completely oppose* to 6 = *completely support*) for negotiations to obtain different outcomes [i.e., achieving peace between Israelis and Palestinians, long-term truce between Hamas and Israel, and achieving peace based on the Arab Peace Initiative (Shikaki and Scheindlin, 2019; Yaar and Rosler, 2019)].

Socio-demographic variables: *Political orientation, gender* and *age* were measured and added to the analysis as covariates. *Political orientation* was measured with a standard self-identifying item for assessing political orientation with regard to security-related issues and the Israeli-Palestinian conflict on a scale ranging from 1 = *strong right* to 7 = *strong left.*

### Results

For means, standard deviations and correlations across all measured variables, see Table 3. To examine our hypotheses, we ran a series of one-way ANOVAs for each of our dependent variables (see means and SDs for each condition in Table 4). Since we found that participants’ political orientation, gender, and age correlated with our dependent variables (see Table 3), and since our conditions were unbalanced in terms of participants’ gender [with 45.1% women to 54.9% men in the IPM condition, and 55.1% women to 44.9% men in the control condition; χ^2^(1) = 4.95, *p* = 0.032; conditions were similar in terms of participants’ political orientation and age, both *p*s > 0.663], we controlled for these background variables throughout the statistical analysis in order to eliminate potential alternative explanations. Not controlling for these variables had no effect on the results (see Supplementary Materials).

**TABLE 3:** Descriptive statistics and bivariate correlations (Study 2).

	*M*	*SD*	1	2	3	4	5	6
1. Support for negotiation	3.75	1	–	–	–	–	–	
2. Acceptance of IPM	3.85	0.49***	1	–	–	–	–	
3. Deliberation	0.00	0.21***	0.21***	1	–	–	–	
4. Political orientation	2.99	0.42***	0.20***	0.04	1	–	–	
5. Age	39.09	0.18***	0.10*	0.05	0.16***	1	–	
6. Gender (0 = *M*, 1 = *F*)	–	0.15**	0.15**	0.04	0.06	0.05	1	

N = 502; *p < 0.05; **p < 0.01; ***p < 0.001.

**TABLE 4:** Descriptive statistics for dependent variables across conditions in Study 2.

	Deliberation M (SD)	Acceptance of IPM M (SD)	Support for negotiation M (SD)
Control (*n* = 265)	−0.39 (0.59)	3.72 (1.19)	3.62 (1.46)
IPM (*n* = 237)	0.43 (0.84)	4.01 (1.22)	3.90 (1.47)

The analysis showed that compared to the control, the IPM condition led to greater deliberation of the information from the videos [*F*(1,497) = 161.90, *p* < 0.001, Cohen’s *d* = 1.13], greater acceptance of the IPM-based message [*F*(1,497) = 7.49, *p* = 0.006, Cohen’s *d* = 0.24], and greater support for negotiations to promote peaceful outcomes [*F*(1,497) = 5.95, *p* = 0.015, Cohen’s *d* = 0.19].

#### Serial Multiple Mediation Structural Model

To assess the hypothesized roles of deliberation of new information and acceptance of the IPM-based message as mediators of the effect of the IPM-based intervention on support for negotiations, we used the AMOS 25 statistical program to conduct Structural Equation Modeling (SEM). To affirm the distinctiveness of the scales, we first advanced a measurement model consisting of factor-loading paths from the latent constructs (i.e., deliberation of new information, acceptance of IPM-based message, support for negotiations) to their manifest indicators and non-directional correlations between the latent variables. The measurement model had good fit to the data [χ^2^(41, *N* = 502) = 147.17, *p* < 0.001; *NFI* = 0.94; *IFI* = 0.96; *CFI* = 0.96; *RMSEA* = 0.07; *SRMR* = 0.05]. Correlations between the constructs corresponded with the ones reported in Table 3. Factor loadings on all latent variables were significant and ranged from 0.58 to 0.91.

In the next stage, we advanced the serial mediation structural model. Experimental condition (0 = control, IPM = 1) was specified as an exogenous variable, predicting deliberation of new information, which, in turn, predicted acceptance of the IPM-based message, which predicted support for negotiations. We also allowed direct paths from condition to acceptance of IPM-based message and support for negotiations, and a direct path from deliberation of new information to support for negotiations. In addition, we controlled for political orientation, sex and age, which were specified as exogenous variables predicting all other variables except experimental condition.

**FIGURE 2:**
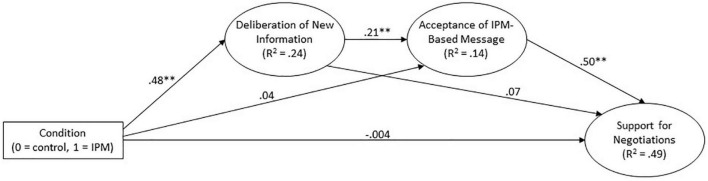
Serial mediation structural model of the effect of the IPM-based intervention on support for negotiation in Study 2. Standardized coefficients are shown. Control variables (political orientation, gender, age), error terms and indicators are not shown. Full information is available in the Supplementary Materials. All coefficients correspond to the arrows beneath them. ^**^p < 0.001.

The structural model and path coefficients can be seen in Figure 2 (control variables are omitted for simplicity; details about their effects can be found in the Supplementary Materials). The model fit the data well [χ^2^(76, *N* = 502) = 279.39, *p* < 0.001; *NFI* = 0.91; *IFI* = 0.93; *CFI* = 0.93; *RMSEA* = 0.07; *SRMR* = 0.05]. Importantly, all the hypothesized paths were significant: Experimental condition had a significant effect on deliberation of new information, which was a significant predictor of acceptance of the IPM-based message, which, in turn, was a significant predictor of support for negotiations. The direct paths from condition to acceptance of the IPM-based message and to support for negotiations were not significant, and neither was the direct path from deliberation of new information to support for negotiations. However, the indirect path from experimental condition to support for negotiations (assessed using bootstrapping with 5000 iterations) was significant {standardized indirect effect = 0.10, 95% confidence interval (CI) [0.04,0.17], *p* = 0.003}, supporting the hypothesized serial mediation.

## Study 3

Following the results of Study 2, we conducted another experiment using the IPM intervention with the aim of replicating the findings and refining the intervention. In the first study, the messages presented in the videos mixed the conflict-supporting narrative themes of delegitimization of the rival and justness of own-goals. The second study was designed to test whether a message containing one of the two themes was more effective than a message containing the other, and how single-theme messages would compare to a message combining the two. To avoid confounding narrative theme with conflict context, the messages in all conditions included examples from two contexts: The Northern Ireland conflict and the French-Algerian war. We hypothesized that all IPM-based interventions would lead to deliberation of the information conveyed, increased acceptance of the IPM-based message, and increased support for negotiations compared to a control group. Since we had no *a priori* hypotheses as to which narrative theme would be most effective, the comparisons between the different IPM-based interventions were exploratory.

### Method

#### Participants

One thousand and eight Israeli-Jews completed the survey through an online surveying company, Midgam (*M*_*age*_ = 38.89, *SD*_*age*_ = 13.18; 50.0% women). In terms of political orientation, the sample resembled the Israeli-Jewish population, as 62.4% were rightists, 23.4% were centrists, and the remaining 14.2% were leftists. In exchange for participation, participants received 4.5 ILS (equivalent to 1.3$). Sensitivity power analysis indicated that our sample size allowed us to detect an effect size of Cohen’s *d* = 0.18, with 80% power when contrasting the control condition with the three IPM conditions.

#### Procedure and Materials

Participants were asked to take part in a study in which they would watch a short video and respond to some questions. They were then randomly assigned to one of four conditions. Participants in the *IPM-delegitimization* condition (*n* = 234) watched a short 1.5-min video containing, in randomized order, two 40-s “How Conflicts End” clips in Hebrew, similar to the ones used in Study 2, both focusing on delegitimization of the rival, with one video using the conflict in Northern Ireland as an example, and the other using the French-Algerian War. Participants in the *IPM-justness* condition (*n* = 246) watched a short 1.5-min video containing, in randomized order, two 40-s “How Conflicts End” clips in Hebrew, focusing on the justness of the ingroup’s goals, using the same two conflicts as examples. Participants in the *IPM-combined* condition (*n* = 248) were asked to watch a short 1.5-min video containing, in a randomized order, one clip focusing on the delegitimization of the rival and a second focusing on justness of the ingroup’s goals. We randomly selected one IPM video from each of the previous two conditions in order to expose each participant to both themes (i.e., delegitimization and justness of goals) and both contexts (i.e., the Northern Ireland conflict, and the French-Algerian war). In the *control* condition, participants (*n* = 280) watched a 1.5-min video, which contained two generic television commercials unrelated to intergroup relations.

After watching the video, participants responded to two multiple choice attention verification questions—one for each video, which were identical to Study 1. Those who responded correctly continued to complete the dependent variables questionnaire, which included the measures detailed below in a fixed order, as well as some additional exploratory measures (for information about the additional measures, see https://osf.io/qr6jn/?view_only=f0dedb3658a24e9c86f351e9ec03a4fc).

#### Measures

*Deliberation of new information* (α = 0.86), *support for negotiation* (α = 0.84), and *socio-demographic variables* were all measured with the exact same items as in Study 2.

*Acceptance of IPM-based message* regarding conflict-supporting narratives and their implications (α = 0.78) was measured using the same items as in Study 2, to which we added one item assessing acceptance of the IPM-based message referring to justness of the ingroup’s goals (i.e., ‘‘Views regarding the absolute justness of societies involved in conflict develop due to conflict-related events.’’).^4^

### Results

For means, standard deviations and correlations across all measured variables, see Table 5. Given that we hypothesized that the IPM intervention would be effective regardless of the specific content of the intervention, to examine our hypotheses, we ran a series of planned Helmert contrasts for each of our dependent variables (see means and SDs for each condition in Table 6). The first contrast (D1) compared the control condition to the three IPM conditions. The second contrast (D2) compared the IPM-combined condition to the two single-themed conditions (IPM-delegitimization and IPM-justness). The third contrast (D3) compared the IPM-delegitimization condition to the IPM-justness condition. Coding in this way allowed us to assess the general effectiveness of the IPM-based messages compared to the control group, which was hypothesized, as well as conduct exploratory comparisons of the effects of different message themes and their combination. Since we found that participants’ political orientation, gender and age correlated with our dependent variables (see Table 5), and to be consistent with the analysis in Study 2, we controlled for these background variables throughout the statistical analysis (there was no bias across conditions in terms of political orientation, age, and gender; all *p*s > 0.442). Not controlling for these variables had no effect on the results (see Supplementary Materials).

**TABLE 5:** Descriptive statistics and bivariate inter-correlations (Study 3).

	*M*	*SD*	1	2	3	4	5	6
1. Support for negotiation	3.88	1.45	1	–	–	–	–	–
2. Acceptance of IPM	3.89	1.17	0.44***	1	–	–	–	–
3. Deliberation	0.00	0.80	0.25***	0.35***	1	–	–	–
4. Political orientation	3.07	1.37	0.46***	0.26***	0.12**	1	–	–
5. Age	38.89	13.18	0.23***	0.12***	0.10**	0.28***	1	–
6. Gender (0 = *M*, 1 = *F*)	–	–	0.08**	0.03	0.08*	0.07*	0.03	1

N = 1008; *p < 0.05; **p < 0.01; ***p < 0.001.

**TABLE 6:** Descriptive statistics for dependent variables across conditions in Study 3.

	Deliberation M (SD)	Acceptance of IPM M (SD)	Support for negotiation M (SD)
Control (*n* = 280)	−0.37 (0.69)	3.52 (1.24)	3.75 (1.49)
IPM-delegitimization (*n* = 234)	0.12 (0.81)	4.07 (1.15)	4.02 (1.43)
IPM-justness (*n* = 246)	0.14 (0.78)	3.96 (1.09)	3.83 (1.45)
IPM-combined (*n* = 248)	0.15 (0.78)	4.07 (1.06)	3.94 (1.43)

#### Deliberation

The analysis yielded a significant effect of the D1 contrast, indicating that deliberation in the three IPM conditions was greater than in the control condition (*b* = 0.51, *SE* = 0.05, *p* < 0.001, 95% CI [0.40,0.61]). The two additional contrasts were not significant (D2: *b* = -0.02, *SE* = 0.06, *p* = 0.743, 95% CI [-0.14,0.10]; D3: *b* = -0.02, *SE* = 0.07, *p* = 0.770, 95% CI [-0.16,0.12]), revealing no differences between the three IPM conditions.

#### Acceptance of the Informative Process Model-Based Message

The analysis yielded a significant effect of the D1 contrast (*b* = 0.51, *SE* = 0.08, *p* < 0.001, 95% CI [0.36,0.67]), indicating greater acceptance of the IPM-based message in the IPM conditions than in the control condition. The two additional contrasts were not significant (D2: *b* = -0.06, *SE* = 0.09, *p* = 0.513, 95% CI [-0.23,0.11]; D3: *b* = 0.11, *SE* = 0.10, *p* = 0.267, 95% CI [-0.09,0.31]), revealing no differences between the three IPM conditions.

#### Support for Negotiation

The analysis yielded a significant effect of the D1 contrast (*b* = 0.18, *SE* = 0.09, *p* = 0.046, 95% CI [0.004,0.36]), indicating greater support for negotiation in the IPM conditions than in the control condition. The two additional contrasts were not significant D2: *b* = -0.02, *SE* = 0.10, *p* = 0.882, 95% CI [-0.21,0.18]; D3: *b* = 0.19, *SE* = 0.12, *p* = 0.104, 95% CI [-0.04,0.42]), revealing no differences between the three IPM conditions.

### Serial Multiple Mediation Structural Model

We tested a serial mediation structural model similar to Study 2, using the AMOS 25 statistical program. As in Study 2, we first advanced a measurement model, which had good fit to the data [χ^2^(41, *N* = 1008) = 266.60, *p* < 0.001; *NFI* = 0.95; *IFI* = 0.96; *CFI* = 0.95; *RMSEA* = 0.07; *SRMR* = 0.07]. The correlations corresponded with the ones reported in Table 5, and factor loadings were all significant and ranged from 0.57 to 0.89.

Since experimental condition was a categorical variable with four levels, we used the three Helmert contrasts described above to represent it. In the serial mediation structural model, the three contrasts were specified as exogenous variables predicting deliberation of new information, which, in turn, predicted acceptance of the IPM-based message, which predicted support for negotiations. We also allowed direct paths from each contrast to acceptance of IPM-based message and support for negotiations, and a direct path from deliberation of new information to support for negotiations. In addition, we controlled for political orientation, sex and age, which were specified as exogenous variables predicting all variables except the three contrasts.

**FIGURE 3:**
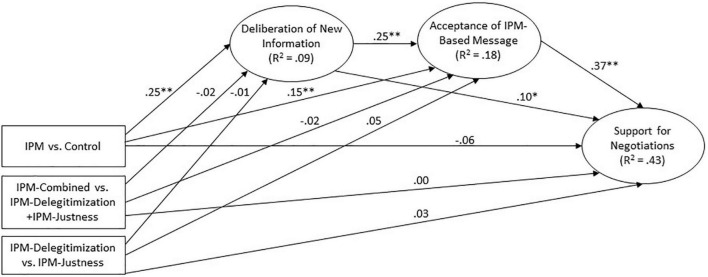
Serial mediation structural model of the effect of the IPM-based intervention on support for negotiation in Study 3. Standardized coefficients are shown. Control variables (political orientation, gender, age), error terms and indicators are not shown. Full information is available in the Supplementary Materials. All coefficients correspond to the arrows beneath them. *p < 0.05 ^**^p < 0.001.

The structural model and path coefficients can be seen in Figure 3 (control variables are omitted for simplicity; details about their effects can be found in the Supplementary Materials). The model fit the data well [χ^2^(117, *N* = 1008) = 569.90, *p* < 0.001; *NFI* = 0.91; *IFI* = 0.92; *CFI* = 0.92; *RMSEA* = 0.06; *SRMR* = 0.05]. Of the contrasts representing the experimental conditions, only the one comparing the IPM conditions to the control group (D1) had any significant effects on the other variables. The contrasts comparing the different IPM messages (D2 and D3) did not have any significant effects. Other hypothesized paths were significant: the D1 contrast had a significant effect on deliberation of new information, which was a significant predictor of acceptance of the IPM-based message, which, in turn, was a significant predictor of support for negotiations. The direct path from the D1 contrast to acceptance of the IPM-based message was significant, but the direct path to support for negotiations was not. There was also a significant direct path from deliberation of new information to support for negotiations. Most importantly, the indirect path from the D1 contrast to support for negotiations (assessed using bootstrapping with 5000 iterations) was significant (standardized indirect effect = 0.11, 95% CI [0.08,0.14], *p* = 0.001), supporting the hypothesized serial mediation.

## General Discussion

Societies involved in intractable conflicts develop functional narratives that serve their basic psychological needs and provide a rationale for their bloody struggle with the enemy (Azar, 1990; Coleman, 2006; Garagozov, 2012; Kriesberg and Dayton, 2016). However, these narratives support the continuation of the conflict with its immense costs to society, and they are often frozen and entrenched. As such, they are destructive to efforts to end the conflict peacefully (Bar-Tal and Halperin, 2011; Hammack, 2011; Garagozov, 2012). Therefore, it is a major challenge to change these narratives through a non-threatening unfreezing process that will eventually advance conflict resolution. Based on the theory of the psychological foundations and dynamics of intractable conflicts (Bar-Tal, 2013, 2019), we expected that awareness and understanding of the socio-psychological processes taking place in intractable conflicts, namely the development of conflict-supporting narratives and their implications, would be associated with recognizing the similarities between an ongoing conflict and other conflicts that have been resolved peacefully.

More specifically, the developed new intervention (IPM) allows the individual to comprehend the important reason for the evolvement of conflict-supporting narratives: to satisfy the fundamental individual and collective needs of society members. This process characterizes every society involved in intractable conflict. Furthermore, the intervention provides unequivocal information that the conflict-supporting narratives are fueling the conflict and contributing to the immense costs that societies pay for its continuation, serving as barriers to its resolution. Finally, the individuals learn, from stories of other societies that successfully resolved their conflict peacefully, that it is possible to change the conflict-supporting narratives with peace-supporting narratives that also satisfy the same human needs. Thus, the intervention opens a new understanding that may unfreeze the frozen conflict supporting narratives, their change and eventually facilitate a construction of new narratives that will enable peaceful resolution of the conflict.

Considering the current studies; in the first study, using representative samples of Palestinians and Israeli Jews we established the hypothesized relationships between awareness of conflict-supporting narratives and their implications, beliefs in the possibility of resolving the Israeli-Palestinian conflict peacefully given its similarity to other conflicts that have been resolved, and support for pursuing peaceful relations between Israelis and Palestinians. In the next two studies conducted with nationwide samples of Israeli Jews we found that short, original, videos that followed the IPM principles led to deliberation of new information, which predicted acceptance of the IPM-based message, which, in turn, predicted support for negotiations. In Study 3, we also found no indications for substantial differences between messages focusing on different themes of conflict-supporting narratives. The studies provide unequivocal evidence that the IPM-based intervention is an effective approach. However, these are the first steps in a long journey of future research to affirm the validity and uniqueness of the IPM-based intervention, its generalization to other conflicts and the exploration of its potential extensions as well as limitations.

### Theoretical and Applied Implications

Our new model and the findings supporting it hold theoretical significance to research dealing with change of beliefs and attitudes in the demanding context of intractable conflicts. Throughout the years, various models of attitude change have been proposed and adapted to this context and to target groups involved in such conflicts (for reviews, see Paluck, 2012; Walton and Wilson, 2018; Bar-Tal and Hameiri, 2020). Most of these models are based on the premise that message contents contradicting existing beliefs lead to change and should be used to induce inconsistency and instigate unfreezing. As we reviewed above, there are a number of alternative approaches that were proposed and tested.

Evaluations of these lines of research indicate that there is no one intervention effective for different contexts, audiences, or type of messages (Bar-Tal and Hameiri, 2020). Each intervention has its own assets and liabilities. The IPM successfully deals with several issues that past models encountered, making it, among other benefits, amenable for scaling up. First, it approaches individuals in a non-threatening manner without creating inconsistencies or confronting them with information that contradicts their current beliefs, which can be met with resistance. On the contrary, by presenting the rationale and legitimacy for the development of conflict-related beliefs, and demonstrating that similar processes have taken place in other societies living in the harsh context of intractable conflicts, IPM-based messages provide individuals with acceptance of their feelings without depicting them in a ridiculous or absurd manner. Furthermore, rather than deceiving participants regarding the source of the message or the intention behind it, these messages are informative, transparent and direct, enabling participants to consciously process the information at a deeper level. Participants also have increased agency as they are encouraged to consider their own beliefs, which can facilitate change that is informed as well as consciously chosen.

Furthermore, Studies 2 and 3 provided initial support for the general effectiveness of IPM-based messages regardless of the specific examples or themes utilized. Study 2 demonstrated the effectiveness of messages including examples from various historical conflicts that ended peacefully, while Study 3 demonstrated that messages focusing on different themes or their combination were almost equally effective. Our findings suggest further practical applications of the IPM approach for changing beliefs in the context of bloody and intractable conflicts. Specifically, the findings show that even under conditions of intractable conflicts, where beliefs are typically deeply entrenched and frozen, change is possible in response to the right message. Moreover, the findings demonstrate the crucial importance of combining the elements of acceptance and change in messages intended to instigate unfreezing and promote conciliatory attitudes in the context of intractable conflict.

### Limitations and Future Directions

The experimental studies of the IPM-based intervention focused on the Israeli Jewish society in the context of the Israeli-Palestinian conflict. Although the Israeli-Palestinian conflict is considered a prototypical example of an intractable conflict, one limitation is our focus on one case study, hence leaving unknown whether the findings generalize to the other parties in this conflict or to other conflicts. The correlations found in Study 1 among Palestinians and our reliance on messages that focused on other conflicts and did not address the Israeli-Palestinian conflict directly, suggest that similar messages may be effective among other parties in this conflict as well as in other contexts of ongoing intractable conflicts. This possibility can be explored in future studies.

A second limitation is our focus on two themes from conflict-supporting narratives (Bar-Tal et al., 2012). Hence, another interesting direction for future research is testing messages relating to other themes within these narratives. Furthermore, since our studies examined the intervention for the first time, we compared them to a control condition. Future studies can compare IPM-based interventions to other intervention techniques.

An additional limitation has to do with the complexity of the IPM-based intervention. The-IPM based message, as depicted in the videos, included numerous components and was compared to a neutral control condition that did not include any of these components, and was in fact unrelated to the conflict at all. Consequently, it is not possible to determine whether all the components of the IPM-based message were necessary to produce the effect, nor whether the combination of the components was more effective than each component alone. Future studies can break down the IPM-based message into its elements and test the necessity and (in)sufficiency of each element in producing the effect. Future studies can also compare the effect of the IPM-based interventions to other interventions that have been developed to change conflict-supporting narratives (Bar-Tal and Hameiri, 2020). This will allow us to determine the relative effectiveness of the IPM-based intervention compared to other interventions, as well as the conditions under which each intervention is more or less effective.

We note that these videos yielded small effect sizes, especially when it came to support for negotiations. However, recent surveys among Jewish-Israelis have shown that support for negotiations with Palestinians to promote a peaceful conflict resolution is at its lowest point in decades (Shikaki et al., 2020; Rosler and Yaar, 2021). Therefore, we believe that the fact that a brief intervention involving watching videos for a few minutes had an effect on Israeli Jews’ support for negotiations, rather than unilateral solutions, is not trivial. As this line of research was the first to examine IPM-based interventions, whether and to what extent the effects we obtained can withhold the detrimental effects of the conflict-related events to which members of societies immersed in intractable conflicts are constantly exposed, remains an open question.

It is important to note that more participants dropped out in the IPM condition(s) (Study 2: 11%; Study 3: between 30 and 33%) compared to the control (Study 2: 2%; Study 3: 19%). There are several potential explanations for why more people dropped out in the IPM conditions than in the control. For example, it might be the case that the videos were less engaging in this condition; that the attention check items were more difficult; that the participants were familiar with the control condition commercials; or that some participants were angered or offended by the IPM videos. Given that in both Studies 2 and 3 we do not have a pre-manipulation measurement, we are unable to assess whether those who dropped out were significantly different from those who completed the studies. However, in both studies, we conducted a preliminary analysis that compared the conditions in terms of the main demographic variables. We found that they were slightly unbalanced only in terms of participants’ gender in Study 2. Correspondingly, we controlled for this background variable to account for this bias.

Finally, our understanding that a society involved in an intractable conflict cannot be taken to therapy based on the acceptance-change principles motivated us to use short videos developed specifically for the current research. However, future studies should seek to extend the generalizability of the IPM principles by using other modes of message presentation besides video-clips, such as vignettes or a recorded lecture. Moreover, based on oral and written reports, we have reason to believe that this method can serve as a basis for developing a curriculum that can be used in classrooms. Anecdotally, the last author used this method in numerous lectures and talks that always led to great interest and deliberation. Therefore, we plan to develop such a curriculum and study its effects rigorously. Lastly, in order to enhance the ecological validity of IPM-based interventions, future studies could include field experiments, as well as examination of the effect of the intervention over a longer period of time.

In sum, we realize that we have embarked on a long and complex journey to meet the challenge of developing an intervention aimed at unfreezing beliefs that perpetuate the continuation of intractable conflicts. In the present studies we have taken the first steps on both conceptual and empirical levels by establishing the new IPM phenomenon. The theory underlying these studies was available for many years (Bar-Tal, 1998, 2013) and only recently we began to apply the accumulated knowledge to practice with the development of the current conceptualization. This first step needs further extensive developments and research in order to translate these ideas to the field. We hope to persuade social scientists that there is a merit in the journey we have begun.

## Data Availability Statement

The datasets presented in this study can be found in online repositories. The names of the repository/repositories and accession number(s) can be found below: https://osf.io/qr6jn/?view_only=f0dedb3658a24e9c86f351e9ec03a4fc.

## Ethics Statement

The studies involving human participants were reviewed and approved by the Ethics Committee of Tel-Aviv University. The patients/participants provided their written informed consent to participate in this study.

## Author Contributions

NR, KS, BH, OW-B, OI, and DB-T contributed to conception and design of the study. NR and BH organized the dataset. KS and BH performed the statistical analysis. NR, KS, BH, OW-B, and DB-T wrote sections of the manuscript. All authors contributed to manuscript revision, read, and approved the submitted version.

## Conflict of Interest

The authors declare that the research was conducted in the absence of any commercial or financial relationships that could be construed as a potential conflict of interest.

## Publisher’s Note

All claims expressed in this article are solely those of the authors and do not necessarily represent those of their affiliated organizations, or those of the publisher, the editors and the reviewers. Any product that may be evaluated in this article, or claim that may be made by its manufacturer, is not guaranteed or endorsed by the publisher.

## Footnotes

1
https://statistics.btselem.org/en/intro/fatalities

2 The complete distribution of responses can be found in the Supplementary Materials.

3 One additional reverse coded item was removed from the final scale as it lowered the scale’s reliability.

4 As in Study 2, the same reverse coded item was removed from the final scale as it lowered the scale’s reliability.

## References

[B1] Al Ramiah A. Hewstone M. (2013). Intergroup contact as a tool for reducing, resolving, and preventing intergroup conflict: evidence, limitations, and potential. *Am. Psychol.* 68:527. 10.1037/a0032603 24128316

[B2] Alkoby A. Halperin E. Tarrasch R. Levit-Binnun N. (2017). Increased support for political compromise in the israeli-palestinian conflict following an 8-week mindfulness workshop. *Mindfulness* 8 1345–1353. 10.1007/s12671-017-0710-5

[B3] Arlo C. (2017). Group therapy and dialectical behavior therapy: an integrative response to a clinical case. *Int. J. Group Psychother.* 67 S13–S23. 10.1080/00207284.2016.1218773 38449265

[B4] Auerbach Y. (2010). “National narratives in a conflict of identity,” in *Barriers to Peace in the Israeli-Palestinian Conflict*, ed. Bar-Siman-Tov Y. (Jerusalem: The Jerusalem Institute for Israel Studies), 99–134.

[B5] Azar E. E. (1990). “Protracted international conflicts: ten propositions,” in *Conflict: Readings in Management and Resolution*, eds Burton J. Dukes F. (London: Palgrave Macmillan), 145–155. 10.1007/978-1-349-21003-9_8

[B6] Bar-Tal D. (1998). Societal beliefs in times of intractable conflict: the Israeli case. *Int. J. Confl. Manag.* 9 22–50. 10.1108/eb022803

[B7] Bar-Tal D. (2013). *Intractable Conflicts: Socio-Psychological Foundations and Dynamics.* Cambridge: Cambridge University Press. 10.1017/CBO9781139025195

[B8] Bar-Tal D. (2019). The challenges of social and political psychology in pursuit of peace: personal account. *Peace Confl. J. Peace Psychol.* 25 182–197. 10.1037/pac0000373

[B9] Bar-Tal D. Halperin E. (2011). “Socio-psychological barriers to conflict resolution,” in *Intergroup Conflicts and Their Resolution: A Socioal Psychological Perspective*, ed. Bar-Tal D. (London: Psychology Press), 217–240. 10.1002/9780470672532.wbepp049

[B10] Bar-Tal D. Hameiri B. (2020). Interventions to change well-anchored attitudes in the context of intergroup conflict. *Soc. Personal. Psychol. Compass* 14:e12534. 10.1111/spc3.12534

[B11] Bar-Tal D. Hammack P. L. (2012). “Conflict, delegitimization, and violence,” in *The Oxford Handbook of Intergroup Conflict*, ed. Tropp L. R. (Oxford: Oxford University Press), 29–52. 10.1093/oxfordhb/9780199747672.013.0003

[B12] Bar-Tal D. Raviv A. (2021). *The Comfort Zone of a Society in Conflict* (In Hebrew). Petach Tikva: Steimatzky.

[B13] Bar-Tal D. Hameiri B. Halperin E. (2021). Paradoxical thinking as a paradigm of attitude change in the context of intractable conflict. *Adv. Exp. Soc. Psychol.* 63:129. 10.1016/bs.aesp.2020.11.003

[B14] Bar-Tal D. Sharvit K. Halperin E. Zafran A. (2012). Ethos of conflict: the concept and its measurement. *Peace Confl. J. Peace Psychol.* 18 40–61. 10.1037/a0026860

[B15] Brodsky B. S. Stanley B. (2013). *The Dialectical Behavior Therapy Primer: How DBT Can Inform Clinical Practice.* Hoboken, NJ: Wiley-Blackwell.

[B16] Brubaker R. Laitin D. D. (1998). Ethnic and nationalist violence. *Annu. Rev. Sociol.* 24 423–452. 10.1146/annurev.soc.24.1.423

[B17] Burton J. (1990). *Conflict: Human Needs Theory.* New York, NY: Macmillan Press. 10.1007/978-1-349-21000-8

[B18] Cacioppo J. T. Petty R. E. (1979). Effects of message repetition and position on cognitive response, recall, and persuasion. *J. Personal. Soc. Psychol.* 37 97–109. 10.1037/0022-3514.37.1.97

[B19] Central Bureau of Statistics (2020). *Statistical Abstracts of Israel 2020.* New Delhi: Central Bureau of Statistics.

[B20] Chen S. Chaiken S. (1997). “The heuristic-systematic model in its broader context,” in *Dual-Process Theories in Social Psychology*, eds Chaiken S. Trope Y. (New York, NY: The guilford Press), 73–96.

[B21] Cheng C. (2009). Dialectical thinking and coping flexibility: a multimethod approach. *J. Personal.* 77 471–494. 10.1111/j.1467-6494.2008.00555.x 19220723

[B22] Clarke-Habibi S. (2005). Transforming worldviews: the case of education for peace in Bosnia and Herzegovina. *J. Transform. Educ*. 3, 33–56.

[B23] Clark J. K. Wegener D. T. (2013). “Message position, information processing, and persuasion: the discrepancy motives model,” in *Advances in Experimental Social Psychology*, Vol. 47 eds Devine P. Plant A. (Cambridge, MA: Academic Press), 189–232. 10.1016/B978-0-12-407236-7.00004-8

[B24] Clark J. K. Wegener D. T. Fabrigar L. R. (2008). Attitudinal ambivalence and message-based persuasion: motivated processing of proattitudinal information and avoidance of counterattitudinal information. *Personal. Soc. Psychol. Bull.* 34 565–577. 10.1177/0146167207312527 18340037

[B25] Coleman P. T. (2003). Characteristics of protracted, intractable conflict: toward the development of a metaframework-I. *Peace Confl*. 9, 1–37.

[B26] Coleman P. T. (2006). Conflict, complexity, and change: a meta-framework for addressing protracted, intractable conflicts—III. *Peace Confl.* 12 325–348. 10.1207/s15327949pac1204_3

[B27] Ditlmann R. K. Samii C. Zeitzoff T. (2017). Addressing violent intergroup conflict from the bottom up? *Soc. Issues Policy Rev.* 11 38–77.

[B28] Eagly A. H. Chaiken S. (1993). *The Psychology of Attitudes.* Fort Worth, TX: Harcourt brace Jovanovich college publishers.

[B29] Eagly A. H. Kulesa P. Brannon L. A. Shaw K. Hutson-Comeaux S. (2000). Why counterattitudinal messages are as memorable as proattitudinal messages: the importance of active defense against attack. *Personal. Soc. Psychol. Bull.* 26 1392–1408. 10.1177/0146167200263007

[B30] Eidelson R. J. Eidelson J. I. (2003). Dangerous ideas: five beliefs that propel groups toward conflict. *Am. Psychol.* 58 182–192. 12772423 10.1037/0003-066x.58.3.182

[B31] Elman M. F. Gerard C. Golan G. Kriesberg L. (2019). *Overcoming Intractable Conflicts: New Approaches to Constructive Transformations.* Lanham, MD: Rowman and Littlefield.

[B32] Evans J. S. B. T. (2008). Dual-processing accounts of reasoning, judgment, and social cognition. *Annu. Rev. Psychol.* 59 255–278. 10.1146/annurev.psych.59.103006.093629 18154502

[B33] Fielding K. S. Hornsey M. J. Thai H. A. Toh L. L. (2020). Using ingroup messengers and ingroup values to promote climate change policy. *Clim. Change* 158 181–199. 10.1007/s10584-019-02561-z

[B34] Garagozov R. Gadirova R. (2019). Narrative intervention in interethnic conflict. *Polit. Psychol.* 40, 449–465.

[B35] Garagozov R. R. (2012). Narratives in conflict: a perspective. *Dyn. Asymmetr. Confl.* 5 101–106. 10.1080/17467586.2012.742955

[B36] Gayer C. C. Landman S. Halperin E. Bar-Tal D. (2009). Overcoming psychological barriers to peaceful conflict resolution: the role of arguments about losses. *J. Confl. Resolut.* 53 951–975. 10.1177/0022002709346257

[B37] Ginges J. Atran S. (2011). War as a moral imperative (not just practical politics by other means). *Proc. R. Soc. B Biol. Sci.* 278 2930–2938. 10.1098/rspb.2010.2384 21325334 PMC3151701

[B38] Girod S. Fassiotto M. Grewal D. Ku M. C. Sriram N. Nosek B. A. (2016). Reducing implicit gender leadership bias in academic medicine with an educational intervention. *Acad. Med.* 91 1143–1150. 10.1097/ACM.0000000000001099 26826068

[B39] Hadjipavlou M. (2007). The cyprus conflict: root causes and implications for peacebuilding. *J. Peace Res.* 44 349–365. 10.1177/0022343307076640

[B40] Halperin E. Cohen-Chen S. Goldenberg A. (2014). Indirect emotion regulation in intractable conflicts: a new approach to conflict resolution. *Eur. Rev. Soc. Psychol.* 25 1–31. 10.1080/10463283.2014.923155

[B41] Halperin E. Porat R. Tamir M. Gross J. J. (2013). Can emotion regulation change political attitudes in intractable conflicts? From the laboratory to the field. *Psychol. Sci.* 24 106–111. 10.1177/0956797612452572 23211565

[B42] Hameiri B. Bar-Tal D. Halperin E. (2019). Paradoxical thinking interventions: a paradigm for societal change. *Soc. Issues Policy Rev.* 13 36–62. 10.1111/sipr.12053

[B43] Hameiri B. Nabet E. Bar-Tal D. Halperin E. (2018). Paradoxical thinking as a conflict-resolution intervention: comparison to alternative interventions and examination of psychological mechanisms. *Personal. Soc. Psychol. Bull.* 44 122–139. 10.1177/0146167217736048 29052459

[B44] Hammack P. L. (2009). Exploring the reproduction of conflict through narrative: israeli youth motivated to participate in a coexistence program. *Peace Conf.* 15 49–74. 10.1080/10781910802589923

[B45] Hammack P. L. (2011). *Narrative and The Politics of Identity: The Cultural Psychology of Israeli and Palestinian Youth.* Oxford: Oxford University Press.

[B46] Hart P. S. Nisbet E. C. (2012). Boomerang effects in science communication: how motivated reasoning and identity cues amplify opinion polarization about climate mitigation policies. *Commun. Res.* 39 701–723. 10.1177/0093650211416646

[B47] Haslam N. Loughnan S. (2014). Dehumanization and infrahumanization. *Annu. Rev. Psychol.* 65 399–423. 10.1146/annurev-psych-010213-115045 23808915

[B48] Hayes S. C. Masuda A. Bissett R. Luoma J. Guerrero L. F. (2004a). DBT, FAP, and ACT: how empirically oriented are the new behavior therapy technologies? *Behav. Ther.* 35 35–54. 10.1016/S0005-7894(04)80003-0

[B49] Hayes S. C. Strosahl K. D. Bunting K. Twohig M. Wilson K. G. (2004b). “What is acceptance and commitment therapy?,” in *A Practical Guide to Acceptance and Commitment Therapy*, eds Hayes S. C. Strosahl K. D. (Berlin: Springer), 3–29. 10.1007/978-0-387-23369-7_1

[B50] Hornsey M. J. Fielding K. S. (2017). Attitude roots and Jiu Jitsu persuasion: understanding and overcoming the motivated rejection of science. *Am. Psychol.* 72 459. 10.1037/a0040437 28726454

[B51] Kahneman D. (2011). *Thinking Fast, Thinking Slow Interpretation.* London: Tavistock.

[B52] Kao D. T. (2011). Message sidedness in advertising: the moderating roles of need for cognition and time pressure in persuasion. *Scand. J. Psychol.* 52 329–340. 10.1111/j.1467-9450.2011.00882.x 21752025

[B53] Katz D. (1960). The functional approach to the study of attitudes. *Public Opin. Q.* 24 163–204. 10.1086/266945

[B54] Kelman H. C. (1958). Compliance, identification, and internalization three processes of attitude change. *J. Confl. Resolut.* 2 51–60. 10.1177/002200275800200106

[B55] Kelman H. C. (2007). “Social-psychological dimensions of international conflict,” in *Peacemaking in International Conflict: Methods and Techniques (rev.ed)*, ed. Zartman I. W. (Washington, DC: United States Institute of Peace), 61–107.

[B56] Kossowska M. Szumowska E. Szwed P. (2020). *The Psychology of Tolerance in Times of Uncertainty.* Milton Park: Routledge. 10.4324/9780367821487

[B57] Kriesberg L. (1993). Intractable conflicts. *Peace Rev.* 5 417–421. 10.1080/10402659308425753

[B58] Kriesberg L. (2005). “Nature, dynamics, and phases of intractability,” in *Grasping the Nettle: Analyzing Cases of Intractable Conflict*, eds Crocker C. A. Osler H. F. Aall P. (Washington, DC: United States Institute of Peace Press), 65–98.

[B59] Kriesberg L. Dayton B. W. (2016). *Constructive Conflicts: From Escalation to Resolution*, 5th Edn. Lanham, MD: Rowman & Littlefield.

[B60] Kruglanski A. W. (2004). *The Psychology of Closed Mindedness.* London: Psychology Press.

[B61] Kruglanski A. W. Webster D. M. (1996). Motivated closing of the mind: “seizing” and “freezing”. *Psychol. Rev.* 103 263–283. 10.1037/0033-295X.103.2.263 8637961

[B62] Kteily N. Bruneau E. Waytz A. Cotterill S. (2015). The ascent of man: theoretical and empirical evidence for blatant dehumanization. *J. Personal. Soc. Psychol.* 109 901–931. 10.1037/pspp0000048 26121523

[B63] Kuhn D. Lao J. (1996). Effects of evidence on attitudes: is polarization the norm? *Psychol. Sci.* 7 115–120. 10.1111/j.1467-9280.1996.tb00340.x

[B64] Kunda Z. (1990). The case for motivated reasoning. *Psychol. Bull.* 108 480–498. 10.1037/0033-2909.108.3.480 2270237

[B65] Lake D. A. Rothchild D. (1998). *The international spread of ethnic conflict: Fear, diffusion, and escalation.* Princeton, NJ: Princeton University Press. 10.1515/9780691219752

[B66] Lederer K. Galtung J. Antal D. (1980). *Human Needs: A Contribution to the Current Debate.* Cambridge, MA: Oelgeschlager, Gunn & Hain.

[B67] Lemmer G. Wagner U. (2015). Can we really reduce ethnic prejudice outside the lab? A meta-analysis of direct and indirect contact interventions. *Eur. J. Soc. Psychol.* 45 152–168. 10.1002/ejsp.2079

[B68] Linehan M. M. (2015). *DBT Skills Training Manual*, 2nd Edn. New York, NY: The Guilford Press.

[B69] Luong K. T. Garrett R. K. Slater M. D. (2019). Promoting persuasion with ideologically tailored science messages: a novel approach to research on emphasis framing. *Sci. Commun.* 41 488–515. 10.1177/1075547019862559

[B70] Maoz I. McCauley C. (2008). Threat, dehumanization, and support for retaliatory aggressive policies in asymmetric conflict. *J. Confl. Resolut.* 52 93–116. 10.1177/0022002707308597

[B71] Marcus E. C. (2014). “Change and conflict: motivation, resistance, and commitment,” in *The Handbook of Conflict Resolution: Theory and Practice*, 3rd Edn, eds Coleman P. T. Deutsch M. Marcus E. C. (San Francisco, CA: Jossey-Bass), 513–532.

[B72] Maslow A. (1970). *Motivation and Personality*, 2nd Edn. Manhattan, NY: Harper & Row.

[B73] Miller A. G. (1993). The attitude polarization phenomenon: role of response measure, attitude extremity, and behavioral consequences of reported attitude change. *J. Personal. Soc. Psychol.* 64:561. 10.1037/0022-3514.64.4.561

[B74] Mitzen J. (2006). Ontological security in world politics: state identity and the security dilemma. *Eur. J. Int. Relat.* 12 341–370. 10.1177/1354066106067346

[B75] Omer H. Elitzur A. C. (2001). What would you say to the person on the roof? A suicide prevention text. *Suicide Life Threat. Behav.* 31 129–139. 10.1521/suli.31.2.129.21509 11459246

[B76] Oren N. (2019). *Israel’s National Identity: The Changing Ethos of Conflict*. Boulder, CO: Lynne Rienner Publishers.

[B77] Paluck E. L. (2009). Reducing intergroup prejudice and conflict using the media: a field experiment in Rwanda. *J. Pers. Soc. Psychol*. 96, 574–587. 19254104 10.1037/a0011989

[B78] Paluck E. L. (2012). “Interventions aimed at the reduction of prejudice and con?ict,” in *The Oxford Handbook of Intergroup Conflict*, ed. Tropp L. R. (Oxford: Oxford University Press), 13–28. 10.1093/oxfordhb/9780199747672.013.0011

[B79] Paolini S. Harwood J. Hewstone M. Neumann D. L. (2018). Seeking and avoiding intergroup contact: future frontiers of research on building social integration. *Soc. Personal. Psychol. Compass* 12:e12422. 10.1111/spc3.12422

[B80] Peterson J. B. Flanders J. L. (2002). Complexity management theory: motivation for ideological rigidity and social conflict. *Cortex* 38, 429–458. 12146676 10.1016/s0010-9452(08)70680-4

[B81] Papadakis Y. Peristianis N. Welz G. (2006). “Divided cyprus: modernity, history, and an island in conflict,” in *Modernity, History, and Cyprus in Divided Cyprus*, eds Papadakis Y. Peristianis N. Welz G. (Bloomington, IN: Indiana University Press), 1–29.

[B82] Papadakis Y. (2008). Narrative, memory and history education in divided Cyprus: a comparison of schoolbooks on the “History of Cyprus.”. *Hist. Mem.* 20 128–148. 10.2979/his.2008.20.2.128

[B83] Rosler N. Yaar E. (2021). *Peace Index, May 2021.* Available online at: https://tau.us20.list-manage.com/track/click?u=e48e18b43a84387efa593e36a&id=7a0328478f&e=2408507b38 (accessed June 1, 2022).

[B84] Rosler N. Hameiri B. Bar-Tal D. Christophe D. Azaria-Tamir S. (2021). Current and future costs of intractable conflicts—can they create attitude change? *Front. Psychol.* 12:681883. 10.3389/fpsyg.2021.681883 34122277 PMC8187953

[B85] Rosler N. Sharvit K. Bar-Tal D. (2018). Perceptions of prolonged occupation as barriers to conflict resolution. *Polit. Psychol.* 39 519–538. 10.1111/pops.12444

[B86] Rumelili B. (2014). *Conflict Resolution and Ontological Security: Peace Anxieties.* Milton Park: Routledge. 10.4324/9781315796314

[B87] Salma M. (2020). Building social cohesion between Christians and Muslims through soccer in post-ISIS Iraq. *Science* 369 866–870. 10.1126/science.abb3153 32792403

[B88] Shikaki K. Scheindlin D. (2019). Role of public opinion in the resilience/resolution of the palestinian-israeli conflict. *Palestine Israel J. Econ.* 24 61–73.

[B89] Shikaki K. Rosler N. Scheindlin D. (2020). *Palestinian-Israeli Pulse: A Joint Poll.* Available online at: https://resolution.tau.ac.il/sites/socsci-english.tau.ac.il/files/media_server/resolution/Summary%20Report_%20English_Joint%20Poll%2026%20Oct%202020.pdf (accessed June 1, 2022).

[B90] Sloman S. A. (2002). “Two systems of reasoning,” in *Heuristics and Biases: The Psychology of Intuitive Judgment*, eds Gilovich T. Griffin D. Kahneman D. (Cambridge: Cambridge University Press), 379–396. 10.1017/CBO9780511808098.024

[B91] Smith M. B. Bruner J. S. White R. W. (1956). *Opinions and Personality.* New York, NY: John Wiley and Sons.

[B92] Taber C. S. Lodge M. (2006). Motivated skepticism in the evaluation of political beliefs. *Am. J. Polit. Sci.* 50 755–769. 10.1111/j.1540-5907.2006.00214.x

[B93] Tetlock P. E. (2003). Thinking the unthinkable: sacred values and taboo cognitions. *Trends Cogn. Sci.* 7 320–324. 10.1016/S1364-6613(03)00135-9 12860191

[B94] Tropp L. R. (2015). “Dismantling an ethos of conflict: strategies for improving intergroup relations,” in *The Social Psychology of Intractable Conflicts*, Vol. 1 eds Halperin E. Sharvit K. (Berlin: Springer), 159–171. 10.1007/978-3-319-17861-5_12

[B95] Turner J. C. Oakes P. J. (1986). The significance of the social identity concept for social psychology with reference to individualism, interactionism and social influence. *Br. J. Soc. Psychol.* 25 237–252.

[B96] Vollhardt J. R. Bilali R. (2015). The role of inclusive and exclusive victim consciousness in predicting intergroup attitudes: findings from Rwanda, Burundi, and DRC. *Polit. Psychol.* 36 489–506. 10.1111/pops.12174

[B97] Walton G. M. Wilson T. D. (2018). Wise interventions: psychological remedies for social and personal problems. *Psychol. Rev.* 125 617–655. 10.1037/rev0000115 30299141

[B98] Xu M. Petty R. E. (2021). Two-sided messages promote openness for morally based attitudes. *Personal. Soc. Psychol. Bull.* 10.1177/0146167220988371 33588648

[B99] Yaar E. Rosler N. (2019). *Peace Index, June 2019.* Available online at: https://gallery.mailchimp.com/e48e18b43a84387efa593e36a/files/41664b0f-4649-4ef7-bd71-7d1ae5cb8f8e/Peace_Index_May_2019_trans.pdf (accessed June 1, 2022).

