# Supplementary material for: The Informative Process Model as a New Intervention for Attitude Change in Intractable Conflicts: Theory and Empirical Evidence
Source: Front Psychol. 2022 Jul 26;13:946410. doi: 10.3389/fpsyg.2022.946410 (PMC9361850; doi:10.3389/fpsyg.2022.946410)
Supplement: Supplementary file 1 [file Data_Sheet_1.docx]

**The Informative Process Model as a New Intervention for Attitude Change in Intractable Conflicts**

Supplementary Materials

**Table S1.** Distribution of responses to the question “Of following possibilities, what do you prefer to do now about Israeli-Palestinian relations?” among Israelis in Study 1

|  | N = 704 (weighted) |
| --- | --- |
| Maintain the situation as it is now | 14.4% |
| Annex the territories or part of the territories^[[1]](#footnote-1)^ | 11.8% |
| A final war against the Palestinians, in which Israel destroys their military capabilities | 19.2% |
| Reach a peace agreement with the Palestinians | 40.8% |
| Other | 5.1% |
| Don’t know \ No answer | 8.7% |

**Table S2.** Distribution of responses to the question “Of following possibilities, what do you prefer to do now about Israeli-Palestinian relations?” among Palestinians in Study 1

|  | N = 1200 |
| --- | --- |
| Maintain the situation as it is now | 12.7% |
| Wage an armed struggle against the Israeli occupation | 33.5% |
| Wage an unarmed struggle against the Israeli occupation | 10.1% |
| Reach a peace agreement with Israel | 37.4% |
| Other | 1.6% |
| Don’t know \ No answer | 4.7% |

*Additional analysis for Study 2: Comparisons between the conditions without political orientation, gender and age as covariates.*

The analysis showed that compared to the control, the IPM condition led to greater deliberation of the information from the videos (*F*(417) = -12.26 , *p* < .001, Cohen’s *d* = 1.10), greater acceptance of the IPM-based message (*F*(500) = -2.23, *p* = .026, Cohen’s *d* = .20), and marginally significantly greater support for negotiations to promote peaceful outcomes (*F*(500) = -1.69, *p* = .091, Cohen’s *d* = .15; see means and SDs for each condition in Table S1).

**Table S3***.* Descriptive statistics for dependent variables across conditions in Study 2.

|  | *Deliberation*  *M (SD)* | *Acceptance of IPM*  *M (SD)* | *Support for negotiation*  *M (SD)* |
| --- | --- | --- | --- |
| Control | -.38 (.59) | 3.74 (1.19) | 3.65 (1.46) |
| IPM | .42 (.84) | 3.98 (1.22) | 3.87 (1.47) |

*Additional analysis for Study 3: Comparisons between the conditions without political orientation, gender and age as covariates.*

See means and SDs for each condition in Table S2.

*Deliberation.* The analysis yielded a significant main effect (*F*(3,1004) = 30.07, *p* < .001, partial *η*^2^ = .08) of experimental condition. Paired comparisons revealed that all IPM conditions led to greater deliberative processing of the new information compared to the control condition (all *p*s < .001), while no significant differences were found between the three IPM conditions (all *p*s > .665).

*Acceptance of the IPM-based message.* The analysis yielded a significant main effect (*F*(3,1004) = 14.69, *p* < .001, partial *η*^2^ = .04) of experimental condition. Paired comparisons revealed that all IPM conditions led to greater acceptance of the IPM-based message compared to the control condition (all *p*s < .001), while no significant differences were found between the three IPM conditions (all *p*s > .277).

*Support for negotiation.* The analysis yielded a marginally significant main effect (*F*(3,1004) = 2.04, *p* = .106, partial *η*^2^ = .01) of experimental condition. Paired comparisons revealed that compared to the control condition, support for negotiation was significantly higher in the IPM-dehumanization condition (*p* = .019). All other comparisons were not significant (all *p*s > .102).

**Table S4***.* Descriptive statistics for dependent variables across conditions in Study 3.

|  | *Deliberation*  *M (SD)* | *Acceptance of IPM*  *M (SD)* | *Support for negotiation*  *M (SD)* |
| --- | --- | --- | --- |
| Control | -.37_a_ (.69) | 3.51_a_ (1.24) | 3.73_a_ (1.49) |
| IPM-dehumanization | .13_b_ (.81) | 4.07_b_ (1.15) | 4.03_b_ (1.43) |
| IPM-just | .14_b_ (.78) | 3.96_b_ (1.09) | 3.84_ab_ (1.45) |
| IPM-combined | .16_b_ (.78) | 4.07_b_ (1.06) | 3.94_ab_ (1.43) |

*Note*: Means not sharing the same subscript within each dependent variable are significantly different from each other at *p* < .05.

*Study 2 serial mediation structural model details*

**Table S5:** Regression weights in the serial mediation structural model of Study 1

| Effect | | | Estimate | S.E. | Standardized Estimate | C.R. | p |
| --- | --- | --- | --- | --- | --- | --- | --- |
| deliberation | <--- | condition | .691 | .067 | .476 | 10.318 | < .001 |
| deliberation | <--- | political orientation | .015 | .023 | .027 | .650 | .516 |
| deliberation | <--- | gender | .116 | .060 | .080 | 1.916 | .055 |
| deliberation | <--- | age | .004 | .002 | .065 | 1.540 | .124 |
| acceptance | <--- | political orientation | .137 | .038 | .183 | 3.588 | < .001 |
| acceptance | <--- | gender | .339 | .101 | .170 | 3.370 | < .001 |
| acceptance | <--- | age | .004 | .004 | .047 | .937 | .349 |
| acceptance | <--- | deliberation | .291 | .085 | .211 | 3.421 | < .001 |
| acceptance | <--- | condition | .085 | .114 | .043 | .753 | .451 |
| negotiation | <--- | condition | -.013 | .137 | -.004 | -.096 | .924 |
| negotiation | <--- | deliberation | .141 | .102 | .066 | 1.379 | .168 |
| negotiation | <--- | political orientation | .405 | .047 | .345 | 8.646 | < .001 |
| negotiation | <--- | gender | .049 | .122 | .016 | .401 | .689 |
| negotiation | <--- | age | .008 | .005 | .071 | 1.841 | .066 |
| negotiation | <--- | acceptance | .778 | .089 | .498 | 8.731 | < .001 |
| deliberation item 1 (standardized) | <--- | deliberation | 1.000 |  | .724 |  |  |
| deliberation item 2 (standardized) | <--- | deliberation | 1.180 | .064 | .853 | 18.526 | < .001 |
| deliberation item 3 (standardized) | <--- | deliberation | 1.118 | .064 | .809 | 17.569 | < .001 |
| deliberation item 4 (standardized) | <--- | deliberation | 1.237 | .064 | .895 | 19.317 | < .001 |
| deliberation item 5 (standardized) | <--- | deliberation | .821 | .064 | .594 | 12.841 | < .001 |
| acceptance item 1 | <--- | acceptance | 1.000 |  | .651 |  |  |
| acceptance item 2 | <--- | acceptance | 1.180 | .100 | .790 | 11.764 | < .001 |
| acceptance item 3 | <--- | acceptance | .921 | .087 | .605 | 10.575 | < .001 |
| negotiation item 1 | <--- | negotiation | .923 | .041 | .857 | 22.415 | < .001 |
| negotiation item 2 | <--- | negotiation | .648 | .047 | .583 | 13.891 | < .001 |
| negotiation item 3 | <--- | negotiation | 1.000 |  | .914 |  |  |

**Table S6:** Covariances and correlations in the serial mediation structural model of Study 1

|  |  |  | Covariance  Estimate | S.E. | Correlation  Estimate | C.R. | p |
| --- | --- | --- | --- | --- | --- | --- | --- |
| political orientation | <--> | age | 2.859 | .790 | .164 | 3.621 | < .001 |
| gender | <--> | age | .299 | .293 | .046 | 1.021 | .307 |
| political orientation | <--> | gender | .039 | .030 | .058 | 1.307 | .191 |

*Study 2 serial mediation structural model without covariates*

**Table S7:** Regression weights in a serial mediation structural model, similar to Study 2 but without covariates

| Effect | | | Estimate | S.E. | Standardized Estimate | C.R. | P |
| --- | --- | --- | --- | --- | --- | --- | --- |
| Deliberation | <--- | condition | .678 | .067 | .469 | 10.087 | < .001 |
| Acceptance | <--- | deliberation | .339 | .087 | .247 | 3.878 | < .001 |
| Acceptance | <--- | condition | .009 | .116 | .005 | .080 | .936 |
| negotiation | <--- | condition | -.074 | .148 | -.024 | -.498 | .618 |
| negotiation | <--- | deliberation | .145 | .111 | .068 | 1.305 | .192 |
| negotiation | <--- | acceptance | .911 | .098 | .589 | 9.339 | < .001 |
| deliberation item 1 (standardized) | <--- | deliberation | 1.000 |  | .723 |  |  |
| deliberation item 2 (standardized) | <--- | deliberation | 1.178 | .064 | .852 | 18.430 | < .001 |
| deliberation item 3 (standardized) | <--- | deliberation | 1.118 | .064 | .808 | 17.504 | < .001 |
| deliberation item 4 (standardized) | <--- | deliberation | 1.236 | .064 | .894 | 19.231 | < .001 |
| deliberation item 5 (standardized) | <--- | deliberation | .820 | .064 | .592 | 12.775 | < .001 |
| acceptance item 1 | <--- | acceptance | 1.000 |  | .647 |  |  |
| acceptance item 2 | <--- | acceptance | 1.174 | .102 | .782 | 11.545 | < .001 |
| acceptance item 3 | <--- | acceptance | .940 | .089 | .613 | 10.546 | < .001 |
| negotiation item 1 | <--- | negotiation | .954 | .046 | .871 | 20.704 | < .001 |
| negotiation item 2 | <--- | negotiation | .659 | .048 | .582 | 13.608 | < .001 |
| negotiation item 3 | <--- | negotiation | 1.000 |  | .899 |  |  |

*Study 3 serial mediation structural model details*

**Table S8:** Regression weights in the serial mediation structural model of Study 2

| Effect | | | Estimate | S.E. | Standardized Estimate | C.R. | p |
| --- | --- | --- | --- | --- | --- | --- | --- |
| deliberation | <--- | D1: IPM condition vs. control | .354 | .047 | .252 | 7.481 | < .001 |
| deliberation | <--- | political orientation | .035 | .016 | .076 | 2.247 | .025 |
| deliberation | <--- | D2: IPM combined vs. delegitimization + justification | -.024 | .050 | -.015 | -.478 | .633 |
| deliberation | <--- | D3: delegitimization vs. justification | -.015 | .059 | -.008 | -.253 | .800 |
| deliberation | <--- | gender | .080 | .041 | .064 | 1.973 | .049 |
| deliberation | <--- | age | .004 | .002 | .086 | 2.556 | .011 |
| acceptance | <--- | D1: IPM condition vs. control | .310 | .068 | .154 | 4.533 | < .001 |
| acceptance | <--- | D2: IPM combined vs. delegitimization + justification | -.045 | .072 | -.020 | -.626 | .532 |
| acceptance | <--- | deliberation | .363 | .056 | .254 | 6.509 | < .001 |
| acceptance | <--- | political orientation | .157 | .023 | .238 | 6.730 | < .001 |
| acceptance | <--- | D3: delegitimization vs. justification | .137 | .084 | .053 | 1.634 | .102 |
| acceptance | <--- | gender | .005 | .058 | .003 | .091 | .927 |
| acceptance | <--- | age | .001 | .002 | .008 | .230 | .818 |
| negotiation | <--- | political orientation | .404 | .033 | .374 | 12.418 | < .001 |
| negotiation | <--- | deliberation | .233 | .075 | .099 | 3.099 | .002 |
| negotiation | <--- | acceptance | .610 | .063 | .372 | 9.678 | < .001 |
| negotiation | <--- | D1: IPM condition vs. control | -.183 | .094 | -.056 | -1.938 | .053 |
| negotiation | <--- | D2: IPM combined vs. delegitimization + justification | .012 | .100 | .003 | .120 | .904 |
| negotiation | <--- | D3: delegitimization vs. justification | .117 | .116 | .027 | 1.001 | .317 |
| negotiation | <--- | gender | .087 | .081 | .029 | 1.080 | .280 |
| negotiation | <--- | age | .010 | .003 | .089 | 3.125 | .002 |
| deliberation item 1 (standardized) | <--- | deliberation | 1.000 |  | .629 |  |  |

Table continues next page…

Table S8 continued…

| Effect | | | Estimate | S.E. | Standardized Estimate | C.R. | p |
| --- | --- | --- | --- | --- | --- | --- | --- |
| deliberation item 2 (standardized) | <--- | deliberation | 1.320 | .063 | .831 | 20.841 | < .001 |
| deliberation item 3 (standardized) | <--- | deliberation | 1.193 | .061 | .751 | 19.445 | < .001 |
| deliberation item 4 (standardized) | <--- | deliberation | 1.380 | .065 | .869 | 21.362 | < .001 |
| deliberation item 5 (standardized) | <--- | deliberation | .985 | .059 | .620 | 16.749 | < .001 |
| acceptance  item 1 | <--- | acceptance | 1.000 |  | .584 |  |  |
| acceptance  item 2 | <--- | acceptance | 1.405 | .079 | .840 | 17.732 | < .001 |
| acceptance  item 3 | <--- | acceptance | 1.298 | .075 | .771 | 17.285 | < .001 |
| acceptance  item 4 | <--- | acceptance | .905 | .064 | .559 | 14.042 | < .001 |
| negotiation  item 1 | <--- | negotiation | 1.000 |  | .886 |  |  |
| negotiation  item 2 | <--- | negotiation | .952 | .031 | .862 | 30.769 | < .001 |
| negotiation  item 3 | <--- | negotiation | .754 | .034 | .649 | 22.156 | < .001 |

**Table S9:** Covariances and correlations in the serial mediation structural model of Study 2

|  |  |  | Covariance  Estimate | S.E. | Correlation  Estimate | C.R. | P |
| --- | --- | --- | --- | --- | --- | --- | --- |
| gender | <--> | age | .208 | .208 | .032 | 1.003 | .316 |
| political orientation | <--> | age | 5.023 | .588 | .279 | 8.541 | < .001 |
| political orientation | <--> | gender | .050 | .022 | .073 | 2.324 | .020 |

*Study 3 serial mediation structural model without covariates*

**Table S10:** Regression weights in a serial mediation structural model, similar to Study 3 but without covariates

| Effect | | | Estimate | S.E. | Standardized Estimate | C.R. | P |
| --- | --- | --- | --- | --- | --- | --- | --- |
| deliberation | <--- | D1: IPM condition vs. control | .358 | .048 | .255 | 7.483 | < .001 |
| deliberation | <--- | D2: IPM combined vs. delegitimization + justification | -.026 | .051 | -.017 | -.510 | .610 |
| deliberation | <--- | D3: delegitimization vs. justification | -.011 | .059 | -.006 | -.191 | .848 |
| acceptance | <--- | D1: IPM condition vs. control | .302 | .070 | .151 | 4.313 | < .001 |
| acceptance | <--- | D2: IPM combined vs. delegitimization + justification | -.037 | .073 | -.017 | -.508 | .611 |
| acceptance | <--- | D3: delegitimization vs. justification | .135 | .086 | .052 | 1.573 | .116 |
| acceptance | <--- | deliberation | .401 | .057 | .281 | 6.996 | < .001 |
| negotiation | <--- | deliberation | .280 | .081 | .122 | 3.436 | < .001 |
| negotiation | <--- | acceptance | .770 | .070 | .479 | 11.005 | < .001 |
| negotiation | <--- | D1: IPM condition vs. control | -.244 | .103 | -.076 | -2.382 | .017 |
| negotiation | <--- | D2: IPM combined vs. delegitimization + justification | .036 | .108 | .010 | .328 | .743 |
| negotiation | <--- | D3: delegitimization vs. justification | .094 | .127 | .022 | .743 | .458 |
| deliberation item 1 (standardized) | <--- | deliberation | 1.000 |  | .630 |  |  |
| deliberation item 2 (standardized) | <--- | deliberation | 1.319 | .063 | .831 | 20.894 | < .001 |
| deliberation item 3 (standardized) | <--- | deliberation | 1.191 | .061 | .751 | 19.478 | < .001 |
| deliberation item 4 (standardized) | <--- | deliberation | 1.378 | .064 | .869 | 21.409 | < .001 |
| deliberation item 5 (standardized) | <--- | deliberation | .983 | .059 | .619 | 16.767 | < .001 |
| acceptance  item 1 | <--- | acceptance | 1.000 |  | .582 |  |  |
| acceptance  item 2 | <--- | acceptance | 1.409 | .080 | .840 | 17.690 | < .001 |
| acceptance  item 3 | <--- | acceptance | 1.304 | .076 | .773 | 17.262 | < .001 |

Table continues next page…

| Table S10 continued…  Effect | | | Estimate | S.E. | Standardized Estimate | C.R. | P |
| --- | --- | --- | --- | --- | --- | --- | --- |
| acceptance  item 4 | <--- | acceptance | .906 | .065 | .559 | 14.015 | < .001 |
| negotiation  item 1 | <--- | negotiation | 1.000 |  | .865 |  |  |
| negotiation  item 2 | <--- | negotiation | .996 | .035 | .882 | 28.744 | < .001 |
| negotiation  item 3 | <--- | negotiation | .777 | .035 | .653 | 21.929 | < .001 |

1. “The territories” is a term commonly used by Israelis to refer to the West Bank and Gaza Strip. [↑](#footnote-ref-1)
